# Carotid Artery Plaque Progression: Proposal of a New Predictive Score and Role of Carotid Intima-Media Thickness

**DOI:** 10.3390/ijerph19020758

**Published:** 2022-01-11

**Authors:** Nicoletta Brunelli, Claudia Altamura, Carmelina Maria Costa, Riccardo Altavilla, Paola Palazzo, Paola Maggio, Marilena Marcosano, Fabrizio Vernieri

**Affiliations:** 1 Headache and Neurosonology Unit, Neurology, Campus Bio-Medico University of Rome, 00128 Rome, Italy; c.altamura@policlinicocampus.it (C.A.); c.costa@unicampus.it (C.M.C.); marilena.marcosano@unicampus.it (M.M.); f.vernieri@policlinicocampus.it (F.V.); 2 Neurology and Stroke Unit Department, ASST Santi Paolo e Carlo, 20142 Milan, Italy; riccardo.altavilla@asst-santipaolocarlo.it; 3 Department of Neurology, Lausanne University Hospital, Rue du Bugnon 46, 1011 Lausanne, Switzerland; ppalazzo@hotmail.it; 4 Neurology Unit, Riviera-Chablais Hospital, Route du Vieux-Séquoia 20, 1847 Rennaz, Switzerland; 5 Neurology Unit, ASST Bergamo Est, 24068 Bergamo, Italy; paolamaggio82@gmail.com

**Keywords:** carotid plaque progression, carotid intima-media thickness, cerebrovascular disease, HAD_2_S score

## Abstract

Background: We aimed to investigate if the carotid intima-media thickness (IMT) at baseline and the HAD_2_S score, composed of the sum of single risk factors (hypertension, age ≥ 75 years, diabetes, dyslipidemia, smoking), were predictive of plaque progression. Methods: We performed a retrospective analysis on real-life prospectively collected data from patients with any detectable carotid plaque at follow up. The plaque score, calculated at baseline (T0) and at a median follow up of 36.6 months (IQR 39.6–34.3) (T3), was defined as 0: no plaque or stenosis < 30%; 1: stenosis in the range 30–49%; 2: in the range 50–69%; 3: in the range 70–99% and 4: occlusion. Carotid IMT was measured at T0 and T3; HAD_2_S score was calculated at baseline. Results: We included 340 patients with a mean age of 69.9 (9.1) years and 25.3% subjects had plaque progression. Individuals with progression had a median HAD_2_S score of 3 (1) while those without progression had 2 (1). Patients with progression had a mean baseline IMT of 0.86 (0.17) while those without progression had 0.77 (0.18) (*p* < 0.0001). A correlation between progression and baseline IMT was found (*p* = 0.002). Conclusion: Baseline IMT could be considered a predictor of progression. Patients with progression had an HAD_2_S score higher than those without evolution.

## 1. Introduction

Atherosclerotic diseases are the leading cause of death worldwide. Cardiovascular diseases, stroke, and myocardial infarction often occur without warning [1]. Hence, primary prevention of atherosclerotic events is crucial to identifying asymptomatic subjects at high risk [2]. Carotid plaque progression is associated with a higher risk of developing vascular events, in particular ipsilateral stroke [3]. The rapid identification of markers of disease progression could have important clinical implications to improve treatment strategies for patients with a higher vascular risk [4]. Doppler Ultrasound is a simple and non-invasive technique widely used to detect the early stages of atherosclerosis in carotid arteries and provides measures on carotid plaques and carotid intima-media thickness (IMT) [5]. Baseline carotid IMT is a marker of early atherosclerosis and a predictor of stroke and myocardial infarction [6,7,8]. In addition, longitudinal changes in carotid IMT and plaque are also used as markers of atherosclerosis progression [1,9,10]. Carotid IMT and specific plaque characteristics such as the hypoechogenicity, ulcerated surface, and carotid stenosis of 91–99% are related to an increased risk of stroke ipsilateral to the Internal Carotid Artery (ICA) stenosis in asymptomatic subjects [10]. Some studies suggest that carotid IMT is a reliable predictor of a new plaque occurrence [11,12]. Moreover, an increased carotid IMT is an independent factor for stenosis progression in patients with asymptomatic moderate (50–69%) ICA stenoses [13]. Although the predictive role of IMT in new plaques formation and in stenosis progression in patients with moderate carotid stenosis is known, currently no data are available about the role of carotid IMT in stenosis progression in subjects with mild or severe carotid stenosis. Previous studies describing the effect of the principal vascular risk factors on plaque progression and dyslipidemia, smoking, and systolic blood pressure are considered long-term predictors of plaque progression [14]. Although the relationship between individual vascular risk factors and plaque formation and progression has already been studied, the cumulative effect of individual risk factors on plaque evolution has not yet been explored. To our knowledge, no data are currently available about a clinical predictive risk score on plaque progression. We aimed to investigate if the carotid IMT at baseline and the novel proposed risk score, composed of the sum of single vascular risk factors (hypertension, age ≥ 75 years, diabetes, dyslipidemia, smoking, i.e., HAD_2_S score), were predictive of plaque progression in patients with or without detectable carotid plaque at baseline.

## 2. Materials and Methods

### 2.1. Study Population

We performed a retrospective analysis on real-life prospectively collected data at the Headache and Neurosonology Unit (Neurology Unit) at the Campus Bio-Medico University of Rome. For this retrospective study, a dataset containing data of 11,369 patients collected for 15 years (from 2005 to 2020) was used. To study plaque progression, we included only asymptomatic patients who had an observation at baseline (T0) and at 3 years of follow up (T3; median of 36.6 months, IQR 39.6–34.3) with any detectable carotid plaque. We excluded subjects who had only had an observation during these years, or who had an observation period different from 3 years and who did not have any detectable carotid plaque at follow up.

Vascular risk factors, plaque score, IMT, Peak Systolic Velocity (PSV), and End Diastolic Velocity (EDV) were investigated in all patients at T0 and at T3. We performed a standardized screening for vascular risk factors. We defined hypertension as a history of high blood pressure, a systolic blood pressure ≥ 140 mmHg, diastolic blood pressure ≥ 90 mmHg, or the use of an antihypertensive; diabetes as a fasting blood glucose level of ≥126 mg/dL or current treatment for diabetes; hypercholesterolemia as a serum total cholesterol level of 200 mg/dL or the use of lipid-lowering medications. Participants were classified as smokers if current smokers or who had quitted smoking in the last five years. We calculated for all patients, at baseline, a new proposed predictive score using the sum of single vascular risk factors (hypertension, age ≥ 75 years, diabetes, dyslipidemia, smoking) defining the acronym “HAD_2_S” score. Each item had a value of 1 for a total score sum of 5.

### 2.2. Ultrasonographic Examination

Carotid arteries were assessed by continuous wave Doppler and Color flow B-mode Doppler ultrasound using high resolution 7.5 MHz transducers (Philips iU22, Bothell, WA, USA). The best images were digitized and stored for central reading and interpretation. The degree of carotid stenosis was established by means of combined criteria considering blood flow velocities as well as morphological characteristics [15]. According to the Mannheim Consensus, carotid plaque was defined as a focal structure protruding into the arterial lumen of at least 0.5 mm or 50% of the surrounding IMT value or showing a thickness > 1.5 mm measured from the media-adventitia interface to the intima-lumen interface [16]. For each segment, the plaque score was defined as 0: no plaque or stenosis <30%; 1: stenosis in the range 30–49%; 2: stenosis in the range 50–69%; 3: stenosis in the range 70–99% and 4: occlusion. Measurements of IMT were performed on the common carotid artery (CCA) over ≈1.5 cm proximal to the flow divider, using a method previously described [17]. A longitudinal image of the distal CCA was acquired with subjects lying in supine position and the head turned 45° to the left or right. IMT was measured at the thickest plaque-free point on the near and far walls with a specially designed computer program. CCA wall thickness was defined as the mean of the maximum wall thickness of the near and far walls on both the left and right side. To measure IMT, a semiautomatic software (QLAB version 8, Philips Medical Systems, Andover, MA, USA) was used to improve measurement reliability and reproducibility [18,19]. Progression plaque was evaluated by a follow-up ultrasound (US) examination performed with the same modalities as at entry and by the same operators involved in the first US assessment. We defined stenosis progression as any change to a higher category of carotid artery stenosis from baseline to a 3-year follow-up.

### 2.3. Statistical Analysis

Demographic and clinical data were analyzed for the entire study cohort. Statistical analyses using SPSS version 25.0 (SPSS Inc., Chicago, IL, USA) were performed for interval variables with *t*-test (expressed as means with SD) or Mann–Whitney tests (medians with interquartile range [IQR]) according to the results of the Kolmogorov–Smirnov test for data distribution. As a priori analysis, non-parametric tests and contingency tables (Chi-square and two-tailed Fisher exact tests) using unadjusted odds ratios (OR) with their 95% confidence intervals (CI) were run to compare variables between patients with and without plaque progression. Thereafter, we ran forced entry binary logistic regression to define which were the independent determinants of plaque progression among variables that significantly differed between the two groups. All tests were bilateral. Statistical significance was set as two-tailed *p* < 0.05.

## 3. Results

**Figure 1:**
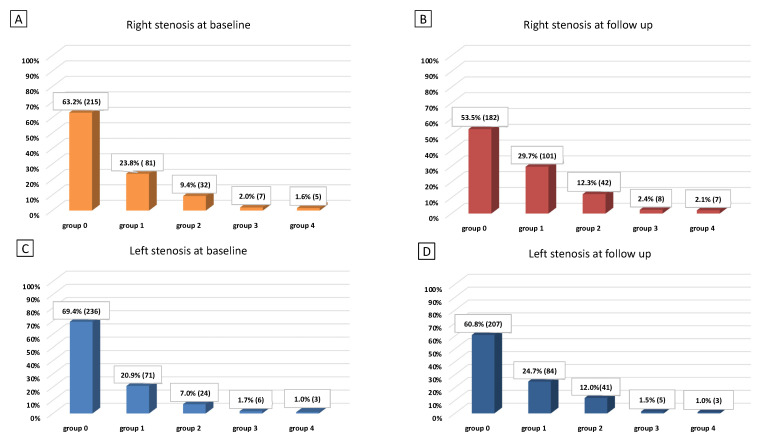
Groups of patients distinguished by right or left carotid stenosis score at baseline (T0) or follow up (T3). (**A**) Groups of patients distinguished by right carotid stenosis score at T0, (**B**) groups of patients distinguished by right carotid stenosis score at T3, (**C**) groups of patients distinguished by left carotid stenosis score at T0, (**D**) groups of patients distinguished by left carotid stenosis score at T3.

**Figure 2:**
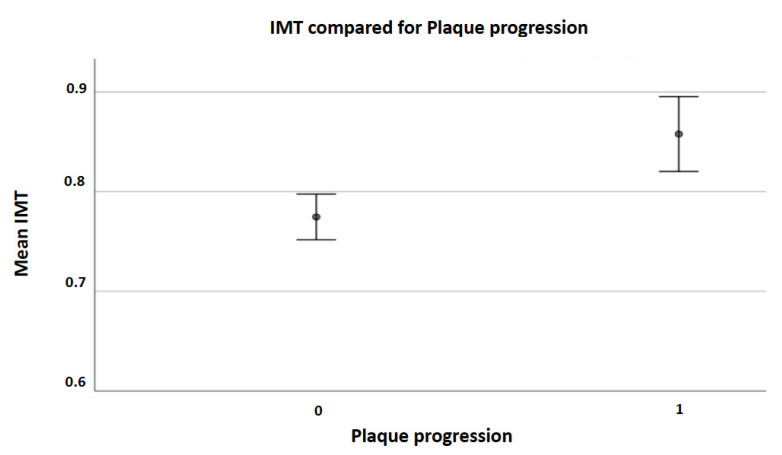
Mean IMT at baseline for each group (non-progression [0] vs. progression [1]). Error bars indicate SD.

We included 340 patients fulfilling the selection criteria with a mean age of 69.9 (9.1) years, 52% of them were men. The median follow-up was 36.6 (39.6–34.3) months. Data were fully available for all subjects. Table 1 displays all demographic characteristics, ultrasound findings, percentage of vascular risk factors, and the median HAD_2_S score at baseline (3% of patients had a HAD_2_S score of 0, 17% had 1, 35% had 2, 33% had 3, 11% had 4 and just 1% of subjects had a HAD_2_S score of 5). Figure 1A shows groups of patients distinguished by right carotid stenosis score at T0, Figure 1B by right carotid stenosis score at T3, Figure 1C by left carotid stenosis score at T0, and Figure 1D by left carotid stenosis score at T3. At T3, 86 (25.3%) subjects had plaque progression; of these 34 (10.0%) in the left side, 39 (11.5%) in the right side, and 13 (3.85%) bilaterally. We registered a small number (5 pts) of cases who underwent carotid endarterectomy or revascularization by angioplasty and stenting. They were advised to undergo intervention as for the presence of an instable plaque (ulceration) or severe stenosis. Thus, we were not able to see the progression of their plaques, so that they were considered as patients who did not progress to occlusion. Individuals with plaque progression had a median HAD_2_S score of 3 (1) while those without progression had a median HAD_2_S score of 2 (1). Patients with stenosis progression had a mean IMT at baseline of 0.86 (0.17) while those without progression had a mean IMT at baseline of 0.77 (0.18) (*p* < 0.0001) (Figure 2). Table 2 displays all demographic characteristics, ultrasound findings, percentage of vascular risk factors, and the median HAD_2_S score at baseline for each group (progression vs. non progression). The post-hoc power calculation for this comparison (progression vs. non progression), assuming an α-error of 0.05, was 100%. We also performed a binary logistic regression analysis of independent determinants of plaque progression showing a significant correlation with mean IMT (*p* = 0.002) (Table 3).

## 4. Discussion

The main finding emerging from our study is the association between the carotid mean IMT at baseline with plaque progression at follow up in a cohort of patients with or without detectable carotid plaques at baseline. Patients with stenosis progression had a mean IMT at baseline of 0.86 (0.17), while those who did not present progression had a mean IMT at baseline of 0.77 (0.18). A positive association between increased baseline carotid IMT and incidence of first-ever carotid plaque has already been described by a meta-analysis of seven prospective studies involving a total of 9,341 patients [20]. Another longitudinal study involving patients with end-stage renal disease described a positive correlation between baseline carotid IMT with the formation rate of new atherosclerotic plaques [21]. Prior studies conducted in individuals at high cardiovascular risk described the association between an increased carotid IMT and incident carotid plaque in a population of 2143 treated hypertensive patients without plaque at baseline [22]. Increased carotid IMT is associated with rapid plaque progression in patients with asymptomatic moderate carotid stenosis (50–69%) at baseline [13]. Increased IMT is associated with risk of stroke in subjects with asymptomatic severe ICA stenosis [10]. An increase in IMT, a higher degree of stenosis, and its progression allow identification of subjects at increased risk of stroke ipsilateral to severe asymptomatic carotid stenosis [10]. Increased carotid IMT is also related with larger brain infarction and clinical severity [18,23] suggesting that IMT could reflect the vulnerability of the atherosclerotic brain to ischemia. To our knowledge, these are the first data demonstrating the association between an increased carotid IMT and plaque progression in all the patients regardless of their stenosis score at baseline. Therefore, our results demonstrate that an increased carotid IMT is predictive of plaque progression not only in individuals naïve for any plaque or with moderate stenosis as previously described but in all patients. This finding suggests that IMT should be considered as a marker to individuate asymptomatic subjects with an increased risk of plaque progression to estimate better their cerebrovascular risk profile and to plan a prevention strategy. Another novel finding of our study is that individuals who presented plaque progression had a median HAD_2_S score at baseline higher than subjects without plaque evolution. This score consists of the sum of the most important five vascular risk factors (hypertension, age, diabetes, dyslipidemia, smoking) involved in atherosclerosis formation and evolution. To our knowledge, no data are available about a clinical predictive score of plaque progression. On the other hand, the effect of each single vascular risk factor on plaque evolution is known. In the Rotterdam study, current smoking habit was the strongest predictor of plaque number increase; the authors also found strong associations for age, total cholesterol, hypertension, and systolic blood pressure [24]. In the Tromsø study, total cholesterol, smoking, and systolic blood pressure were the most robust predictors of total plaque area progression [14]. Current cigarette smoking was a strong independent predictor of carotid plaque progression across ethnicities [25]. In the REFINE study, the authors found a relationship between LDL level, male sex, waist circumference, former smoking habit and physical activity, and new carotid plaque formation [5]. Moreover, some studies demonstrated that subjects with metabolic syndrome are at increased risk for progressive carotid atherosclerosis and coronary heart disease [26]. Although the relationship between individual vascular risk factors and plaque formation and progression is clear, the cumulative effect of individual vascular risk factors on plaque evolution has not yet been explored. The proposal of our new predictive clinical score (HAD_2_S score) aims to explore the influence that the sum of single vascular risk factors may have on plaque progression. Individuals with plaque progression had a median HAD_2_S score of 3 (1) while subjects without progression had a median HAD_2_S score of 2 (1). This result suggests that the presence of three vascular risk factors is enough to influence plaque progression. Simple calculation of this novel HAD_2_S score in all patients with or without carotid plaque at baseline could allow prediction of new plaque genesis or evolution, thus allowing an immediate treatment of the vascular risk factors involved.

This study may have some limitations as we did not have complete data for all subjects included in the study. Some parameters (i.e., treatment, different drugs taken) were not recorded for the entire follow-up, as some subjects were outpatients undergoing duplex scan examination and not clinically followed at our Unit. Hence, we limited our analysis to complete parameters useful for the aim of our study. Finally, it should be noted that we included five patients who underwent intervention due to the presence of an instable plaque (ulceration) or severe stenosis, and that did not present progression of their plaque.

## 5. Conclusions

In conclusion, our results demonstrate that carotid IMT could be considered as a predictor of plaque progression in asymptomatic patients with or without detectable carotid plaques. Further to this, individuals who presented a stenosis progression in our cohort had a median HAD_2_S score at baseline higher than subjects without plaque evolution.

## Figures and Tables

**Table 1:** Demographic characteristics, ultrasound findings, percentage of vascular risk factors and the median HAD_2_S score at baseline.

Patients (*N* = 340)		
*Demographic characteristics*		
Follow up, months (median, IQR)	36.6	39.6–34.3
Age, years (mean, SD)	69.9	9.1
Sex (*n*, % of males)	176	51.8%
		
*Risk factors* (*n*, %)		
Hypertension	275	80.9%
Hyperlipidemia	238	70.0%
Diabetes	79	23.2%
Smoking	45	13.2%
		
*Ultrasound findings*		
IMT (mean, SD)	0.80	0.18
Right ICA PSV (median, IQR)	87	39
Right ICA EDV (median, IQR)	26	14
Left ICA PSV (median, IQR)	90	42
Left ICA EDV (median, IQR)	27	13
		
HAD_2_S score (median, IQR)	2	1

**Table 2:** Demographic characteristics, ultrasound findings, percentage of vascular risk factors and the median HAD_2_S score at baseline for each group (non-progression vs. progression).

	Non-Progression *N* = 254	Progression *N* = 86	*p* Value
Follow up, months (median, IQR)	36.2 (5.2)	37.2 (6.5)	0.104
Age, years (mean, SD)	69.2 (9.2)	71.9 (8.4)	0.012
Sex (*n*, % of males)	126 (49.6)	50 (58.1)	0.212
			
*Risk factors, n* (%)			
Hypertension	201 (79.1)	74 (86.0)	0.204
Hyperlipidemia	179 (70.5)	59 (68.6)	0.786
Diabetes	54 (21.3)	25 (29.1)	0.142
Smoking	32 (12.6)	13 (15.1)	0.582
			
Ultrasound findings *			
IMT (mean, SD)	0.77 (0.18)	0.86 (0.17)	<0.001
Right ICA PSV (median, IQR)	85 (38)	93 (44)	0.063
Right ICA EDV (median, IQR)	26 (15)	26 (11)	0.808
Left ICA PSV (median, IQR)	88 (39)	95.5 (46)	0.036
Left ICA EDV (median, IQR)	27 (13)	27 (15)	0.822
			
*HAD_2_S score*	2 (1)	3 (1)	0.032

* At T3, 86 (25.3%) subjects had plaque progression; of these, 34 (10.0%) in the left side, 39 (11.5%) in the right side and 13 (3.85%) bilaterally.

**Table 3:** Binary logistic regression analysis of independent determinants of plaque progression.

	B	S.E.	Wald	Sig.	ODDs Ratio	95% CI for EXP(B)	
						Lower	Upper
Age T0	0.012	0.017	0.500	0.479	1.012	0.980	1.045
Left PSV T0	0.004	0.002	2.985	0.084	1.004	0.999	1.009
Mean IMT	2.402	0.758	10.038	0.002	11.049	2.500	48.840
HAD_2_S score	0.174	0.144	1.467	0.226	1.190	0.898	1.577
Constant	−4.698	1.174	16.004	0.000	0.009		

## Data Availability

Anonymized data will be shared on request from any qualified investigator.

## References

[1] Huang L.C., Lin R.T., Chen C.F., Chen C.H., Hank Juo S.H., Lin H.F. (2016). Predictors of carotid intima-media thickness and plaque pro-gression in a chinese population. J. Atheroscler. Thromb..

[2] Greenland P., Abrams J., Aurigemma G.P., Bond M.G., Clark L.T., Criqui M.H., CrouseIII J.R., Friedman L., Fuster V., Herrington D.M. (2000). Prevention Conference V: Beyond secondary prevention: Identifying the high-risk patient for primary prevention: Noninvasive tests of atherosclerotic burden: Writing Group III. Circulation.

[3] Balestrini S., Lupidi F., Balucani C., Altamura C., Vernieri F., Provinciali L., Silvestrini M. (2013). One-Year Progression of Moderate Asymptomatic Carotid Stenosis Predicts the Risk of Vascular Events. Stroke.

[4] Mackinnon A.D., Jerrard-Dunne P., Sitzer M., Buehler A., Von Kegler S., Markus H.S. (2004). Rates and determinants of site-specific progression of carotid artery intima-media thickness: The carotid atherosclerosis progression study. Stroke.

[5] Sturlaugsdottir R., Aspelund T., Bjornsdottir G., Sigurdsson S., Thorsson B., Eiriksdottir G., Gudnason V. (2018). Predictors of carotid plaque progression over a 4-year follow-up in the Reykjavik REFINE-study. Atherosclerosis.

[6] Hollander M., Hak A.E., Koudstaal P.J., Bots M.L., Grobbee D.E., Hofman A., Witteman J.C.M., Breteler M.M.B. (2003). Comparison between measures of atherosclerosis and risk of stroke: The Rotterdam study. Stroke.

[7] Rosvall M., Janzon L., Berglund G., Engström G., Hedblad B. (2005). Incidence of stroke is related to carotid IMT even in the absence of plaque. Atherosclerosis.

[8] Polak J.F., Pencina M.J., Meisner A., Pencina K.M., Brown L.S., Wolf P.A., D’Agostino Sr R.B. (2010). Associations of carotid artery intima-media thickness (IMT) with risk factors and prevalent cardiovascular disease: Comparison of mean common carotid artery IMT with maximum internal carotid artery IMT. J. Ultrasound Med..

[9] Silvestrini M., Pasqualetti P., Baruffaldi R., Catani S., Tibuzzi F., Altamura C., Bartolini M., Provinciali L., Vernieri F. (2005). Markers of lacunar stroke in patients with moderate internal carotid artery stenosis. J. Neurol..

[10] Silvestrini M., Altamura C., Cerqua R., Pasqualetti P., Viticchi G., Provinciali L., Paulon L., Vernieri F. (2013). Ultrasonographic Markers of Vascular Risk in Patients with Asymptomatic Carotid Stenosis. Br. J. Pharmacol..

[11] Zureik M., Ducimetière P., Touboul P.J., Courbon D., Bonithon-Kopp C., Berr C., Magne C. (2000). Common carotid intima-media thickness predicts occurrence of carotid atherosclerotic plaques longitudinal results from the aging vascular study (EVA) study. Arterioscler. Thromb. Vasc. Biol..

[12] Von Sarnowski B., Lüdemann J., Völzke H., Dörr M., Kessler C., Schminke U. (2010). Common carotid intima-media thickness and framingham risk score predict incident carotid atherosclerotic plaque formation: Longitudinal results from the study of health in Pomerania. Stroke.

[13] Diomedi M., Scacciatelli D., Misaggi G., Balestrini S., Balucani C., Sallustio F., Di Legge S., Stanzione P., Silvestrini M. (2013). Increased Common Carotid Artery Wall Thickness Is Associated with Rapid Progression of Asymptomatic Carotid Stenosis. J. Neuroimaging.

[14] Herder M., Johnsen S.H., Arntzen K.A., Mathiesen E.B. (2012). Risk factors for progression of carotid intima-media thickness and total plaque area: A 13-year follow-up study: The Tromsø study. Stroke.

[15] De Bray J., Glatt B. (1995). Quantification of Atheromatous Stenosis in the Extracranial Internal Carotid Artery. Cerebrovasc. Dis..

[16] Touboul P.-J., Hennerici M.G., Meairs S., Adams H., Amarenco P., Bornstein N., Csiba L., Desvarieux M., Ebrahim S., Hernandez R.H. (2012). Mannheim Carotid Intima-Media Thickness and Plaque Consensus (2004–2006–2011). Cerebrovasc. Dis..

[17] O’Leary D.H., Polak J.F., Kronmal R.A., Manolio T.A., Burke G.L., Wolfson S.K. (1999). Carotid-Artery Intima and Media Thickness as a Risk Factor for Myocardial Infarction and Stroke in Older Adults. N. Engl. J. Med..

[18] Silvestrini M., Cagnetti C., Pasqualetti P., Albanesi C., Altamura C., Lanciotti C., Bartolini M., Mattei F., Provinciali L., Vernieri F. (2010). Carotid wall thickness and stroke risk in patients with asymptomatic internal carotid stenosis. Atherosclerosis.

[19] Touboul P.-J., Hennerici M., Meairs S., Adams H., Amarenco P., Bornstein N., Csiba L., Desvarieux M., Ebrahim S., Fatar M. (2006). Mannheim Carotid Intima-Media Thickness Consensus (2004–2006). Cerebrovasc. Dis..

[20] Tschiderer L., Klingenschmid G., Seekircher L., Willeit P. (2020). Carotid intima-media thickness predicts carotid plaque development: Meta-analysis of seven studies involving 9341 participants. Eur. J. Clin. Investig..

[21] Benedetto F.A., Tripepi G., Mallamaci F., Zoccali C. (2008). Rate of Atherosclerotic Plaque Formation Predicts Cardiovascular Events in ESRD. J. Am. Soc. Nephrol..

[22] Izzo R., Stabile E., Esposito G., Trimarco V., Laurino F.I., Rao M.A.E., De Marco M., Losi M.A., De Luca N.T., Trimarco B. (2015). Development of new atherosclerotic plaque in hyper-tensive patients: An observational registry study fromthe Campania-Salute network. J. Hypertens..

[23] Heliopoulos I., Papaoiakim M., Tsivgoulis G., Chatzintounas T., Vadikolias K., Papanas N., Piperidou C. (2009). Common carotid intima media thickness as a marker of clinical severity in patients with symptomatic extracranial carotid artery stenosis. Clin. Neurol. Neurosurg..

[24] Van der Meer I.M., Del Sol A.I., Hak A.E., Bots M.L., Hofman A., Witteman J.C.M. (2003). Risk factors for progression of atherosclerosis measured at multiple sites in the arterial tree: The Rotterdam study. Stroke.

[25] Tattersall M.C., Gassett A., Korcarz C.E., Gepner A.D., Kaufman J.D., Liu K.J., Astor B.C., Sheppard L., Kronmal R.A., Stein J.H. (2014). Predictors of carotid thickness and plaque pro-gression during a decade: The multi-ethnic study of atherosclerosis. Stroke.

[26] Bonora E., Kiechl S., Willeit J., Oberhollenzer F., Egger G., Bonadonna R.C., Muggeo M. (2003). Carotid atheroscle-rosis and coronary heart disease in the metabolic syndrome: Prospective data from the Bruneck study. Diabetes Care.

